# Differential Prognosis and Response of Denovo vs. Secondary Muscle-Invasive Bladder Cancer: An Updated Systematic Review and Meta-Analysis

**DOI:** 10.3390/cancers13102496

**Published:** 2021-05-20

**Authors:** Mario Pones, David D’Andrea, Keiichiro Mori, Mohammad Abufraj, Marco Moschini, Eva Comperat, Shahrokh F. Shariat

**Affiliations:** 1 Department of Urology, Medical University of Vienna, 1090 Vienna, Austria; david.dandrea@meduniwien.ac.at (D.D.); sfshariat@gmail.com (S.F.S.); 2 Department of Urology, The Jikei University School of Medicine, Tokyo 3-25-8, Japan; morikeiichiro29@gmail.com; 3 Department of Urology, University of Jordan, Amman 11942, Jordan; dr.abufaraj@gmail.com; 4 Department of Urology, Luzerner Kantonsspital, 6000 Luzern, Switzerland; marco.moschini87@gmail.com; 5 Department of Pathology, Medical University of Vienna, 1090 Vienna, Austria; eva.comperat@meduniwien.ac.at; 6 Comprehensive Cancer Center, Department of Urology, Medical University of Vienna, 1090 Vienna, Austria; 7 Institute of Urology and Reproductive Health, Imperial Moscow Sechenov First Moscow State Medical University, 119992 Moscow, Russia; 8 Department of Urology, Weill Cornell Medical College, New York, NY 10065, USA; 9 Department of Urology, University of Texas Southwestern, Dallas, TX 5323, USA; 10 Karl Landsteiner Institute of Urology and Andrology, 1090 Vienna, Austria; 11 Department of Urology, Second Faculty of Medicine, Charles University, 150 06 Prague, Czech Republic; 12 European Association of Urology Research Foundation, 6803 Arnhem, The Netherlands

**Keywords:** primary MIBC, secondary MIBC, outcome, neoadjuvant chemotherapy, OS, CSS, PFS

## Abstract

**Simple Summary:**

Bladder cancer is one of the leading causes of death worldwide. About 75% of patients initially present with non-muscle-invasive disease, while the rest presents with primary muscle-invasive disease. Up to a third of non-muscle-invasive bladder cancers progresses into secondary muscle-invasive bladder cancer. Little is known about clinical outcomes after upfront neoadjuvant cisplatin-based chemotherapy and subsequent radical cystectomy for secondary muscle-invasive bladder cancer compared to primary muscle-invasive bladder cancer. Here, we systematically reviewed the current literature evaluate oncological outcomes between primary and secondary muscle-invasive bladder cancer.

**Abstract:**

To evaluate oncological outcomes of primary versus secondary muscle-invasive bladder cancer treated with radical cystectomy. Medline, Embase, Scopus and Cochrane Library were searched for eligible studies. Hazard ratios for overall survival (OS), cancer specific survival (CSS) and progression free survival (PFS) were calculated using survival data extracted from Kaplan-Meier curves. A total of 16 studies with 5270 patients were included. Pooled analysis showed similar 5-year and 10-year OS (HR 1, *p* = 0.96 and HR 1, *p* = 0.14) and CSS (HR 1.02, *p* = 0.85 and HR 0.99, *p* = 0.93) between primMIBC and secMIBC. Subgroup analyses according to starting point of follow-up and second-look transurethral resection revealed similar results. Subgroup analyses of studies in which neoadjuvant chemotherapy was administered demonstrated significantly worse 5-year CSS (HR 1.5, *p* = 0.04) but not 10-year CSS (HR 1.36, *p* = 0.13) in patients with secMIBC. Patients with secMIBC had significantly worse PFS at 5-year (HR 1.41, *p* = 0.002) but not at 10-year follow-up (HR 1.25, *p* = 0.34). This review found comparable oncologic outcomes between primMIBC and secMIBC patients treated with RC regarding OS and CSS. Subgroup analysis showed worse 5-year CSS but not 10-year CSS for neoadjuvant chemotherapy in the secMIBC group. Prospective clinical trials incorporating molecular markers, that allow precise risk stratification of secMIBC and further research uncovering underlying molecular and clinical drivers of the heterogeneous group of secMIBC is needed.

## 1. Introduction

Bladder cancer ranks as the ninth most common cancer worldwide with an estimated yearly incidence of about 430,000 new cases, and it ranks 13th regarding yearly cancer mortality [1]. Initially, approximately 75% of patients present with non-muscle-invasive bladder cancer (NMIBC), while the rest present with muscle-invasive bladder cancer (MIBC) or metastasis [2]. In NMIBC tumor recurrence is rather common and up to 30% in the high-risk group (all T1 high-grade without carcinoma in situ [CIS] and all CIS patients) [3] will progress to MIBC [4], despite adequate initial treatments [5]. 

Radical cystectomy (RC) with or without neoadjuvant cisplatin-based combination chemotherapy, when possible, is the standard management of patients with MIBC. About 10–15% of patients with MIBC are initially diagnosed with NMIBC that progressed to MIBC (secondary MIBC = secMIBC), while the remaining patients present with primary MIBC (primMIBC). There is conflicting evidence regarding the differential clinical outcomes of secMIBC after radical cystectomy compared to primMIBC. The question arises if there is a difference in survival between the two and if this is the case could it help physicians to optimize timing of RC in patients with secMIBC. While some studies reported worse survival outcomes of secMIBC compared to primMIBC [4,6,7,8] others did not [9,10,11,12,13,14,15,16,17,18]. In patients with NMIBC indications for RC currently represent a controversial issue. Favorable long-term outcomes are reported in the literature for timely RC in patients with recurrent T1 tumor stage and those with therapy-refractory disease [14,19]. The same is true for the likelihood of response to neoadjuvant cisplatin-based combination chemotherapy [20]. These findings suggest that the prognosis between secMIBC and primMIBC should be explored further to help guide decision making regarding intensity and type of therapy. 

Hence, we conducted a systematic review of the current literature to compare the survival of secMIBC to primMIBC. In addition, we assessed their differential response to neoadjuvant chemotherapy.

## 2. Materials and Methods

### 2.1. Literature Search

This study was conducted according to the PRISMA Statement [21]. A comprehensive electronic search of the following databases was performed: Medline (Ovid), Embase (Ovid), Scopus and the Cochrane Library. In November 2020 the last search was conducted. Language or publication status restrictions were not imposed. The subsequent keywords were used: “bladder cancer”, “bladder tumor”, “urinary bladder neoplasm”, “bladder carcinoma”, “bladder malignancy”, “muscle invasion”, “muscle-invasive”, “cystectomy” and “radical cystectomy”. The completeness of our literature research was ensured by reviewing the references off retrieved articles related to the study topic and cross referencing. Patients presenting initially with muscle-invasiveness are described as primMIBC, those with a former diagnosis of NMIBC as secMIBC. 

### 2.2. Inclusion and Exclusion Criteria

#### 2.2.1. Inclusion Criteria: Studies

1. Conducted in patients diagnosed with bladder cancer.
2. That assessed the prognostic differences between patients with primMIBC and
3. Those with secMIBC who have undergone RC with or without NAC.
4. With no less than 10 patients in each group.
5. That directly reported the hazard ratio (HR) with 95% confidence interval (CI) or in which the reported data allow for calculation of the HR were included in the analysis.

#### 2.2.2. Exclusion Criteria

Letters to the editor, review papers, replies, book chapters, commentaries, case reports and editorials as well as studies that do not include the histological confirmation of bladder cancer were excluded.

The retrieved studies were carefully checked for duplications. When outcomes for the same patient population were reported from more than one publication the most recent and complete study was analyzed. Inconsistencies were resolved via discussion with co-investigators.

### 2.3. Data Extraction

The following data were extracted by two independent investigators: date of publication, author’s last name, year of publication, country, study design, period of patient recruitment and population size, age, sex and follow-up period.

### 2.4. Methodological Quality Assessment

To evaluate the methodological quality of the studies the Newcastle-Ottawa Scale (NOS) (http://www.ohri.ca/programs/clinical_epidemiology/oxford.asp, accessed on: 5 December 2020) was used. Studies are assessed using a star rating system based on selection of study subjects (maximum four stars), comparability of study groups (maximum two stars) and assessment of the outcome (maximum three stars). Since no standardized validated criteria exist, studies rated with seven or more stars were considered to be of high quality.

### 2.5. Statistical Analysis

First, we extracted survival data from the Kaplan-Meier curves. Second, obtained raw data were used to calculate HR and the corresponding 95% CI for overall survival (OS), cancer-specific survival (CSS) and progression-free survival (PFS) using the Cox proportional hazard function described by previous methods [22]. To assess the heterogeneity across the included studies, the chi-square-based Cochrane Q-test was used, with *p* < 0.1 indicating heterogeneity across studies. The magnitude of the study heterogeneity was assessed with the *I*^2^. Heterogeneity was considered significant at *I*^2^ > 50% or chi-square *p* value < 0.1. In this case a random-effects model was applied. Otherwise, a fixed-effect models was applied. Funnel plots were used to assess the publication bias.

Statistical analysis was conducted using RStudio Version 1.3.1993 (Boston, MA, USA) (packages: devtools, reconstructKM, readxl, dplyr, survival), Review Manager Software RevMan version 5.4.1. (The Cochrane Collaboration 2020, London, UK) and Engauge Digitizer software version 2.4.1.

## 3. Results

### 3.1. Study Characteristics

**Figure 1:**
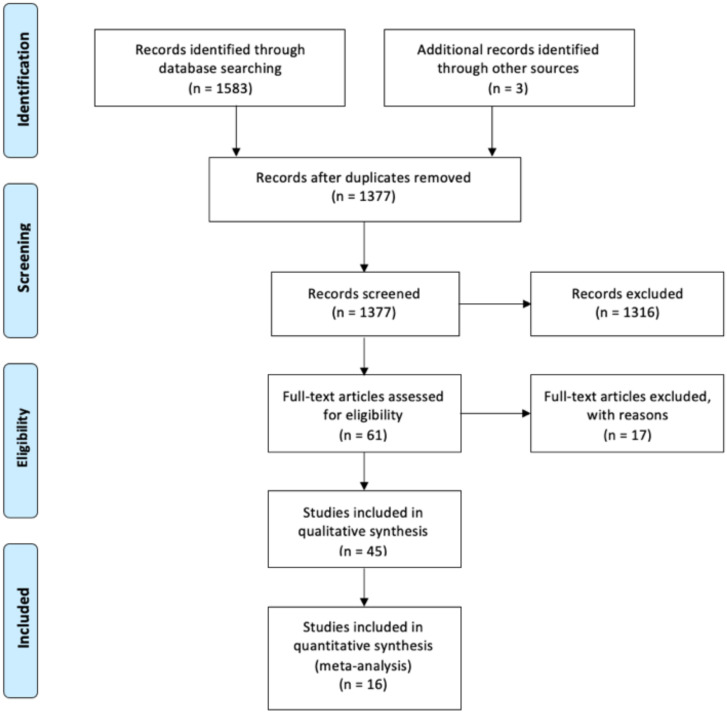
PRISMA 2009 flow diagram.

A total of 1475 studies were identified through the electronic search. The selection process is illustrated in Figure 1. A total of 5270 patients from 16 studies were finally included [4,6,7,8,9,10,11,12,13,14,15,16,17,18,23,24]. Baseline extracted data from the 16 studies are outlined in Table 1. All studies were retrospective cohort studies published between 2002 and 2018. Mean number of patients was 329.4 (range 55–1150). In all studies the “time-to-event” analyses started at the time of RC except for the one conducted by Schrier, which started calculation of survival from the time of MIBC diagnosis and not RC. Therapy of NMIBC consisted of transurethral resection of bladder tumor (=TURBT) and adjuvant instillation therapy. De Vries et al. [15] reported that patients underwent TURBT with or without intravesical instillation before development of muscle-invasiveness occurred. The studies conducted by Kotb et al. [9] and by Kayama et al. [11] provided no data whether intravesical instillation treatment of patients diagnosed with secMIBC was administered.

### 3.2. Patients’ Characteristics

Patient characteristics are summarized in Table 2. Overall, 3686 had primMIBC and 1541 secMIBC. Among the 5270 included patients 79% were male (*n* = 4175) and 21% were female (*n* = 1051). The mean age of the patients in primMIBC was 65.2y, while that in secMIBC was 66.3y.

### 3.3. Overall Survival

**Figure 2:**
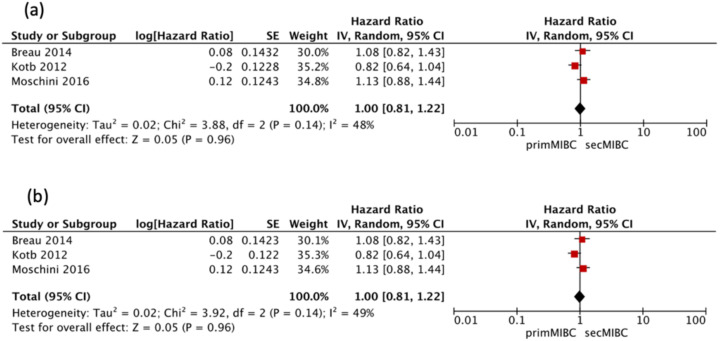
(**a**) Forest Plot for 5-year OS after RC, (**b**) Forrest Plot for 10-year OS after RC.

Three studies [4,7,9] were available for HR calculation of OS between primMIBC and secMIBC. Pooled analysis showed no significant differences in 5-year OS (pooled HR 1, 95% CI 0.81–1.22, *p* = 0.96) without significant heterogeneity between studies (Chi^2^ = 3.88, *I*^2^ = 48%, *p* = 0.14) (Figure 2A). Pooled analysis of the same studies showed no significant difference in 10-year OS (pooled HR 1, 95%CI 0.81–1.22, *p* = 0.96), without significant heterogeneity between studies (Chi^2^ = 3.92, *I*^2^ = 49%, *p* = 0.14) (Figure 2B).

### 3.4. Cancer-Specific Survival

**Figure 3:**
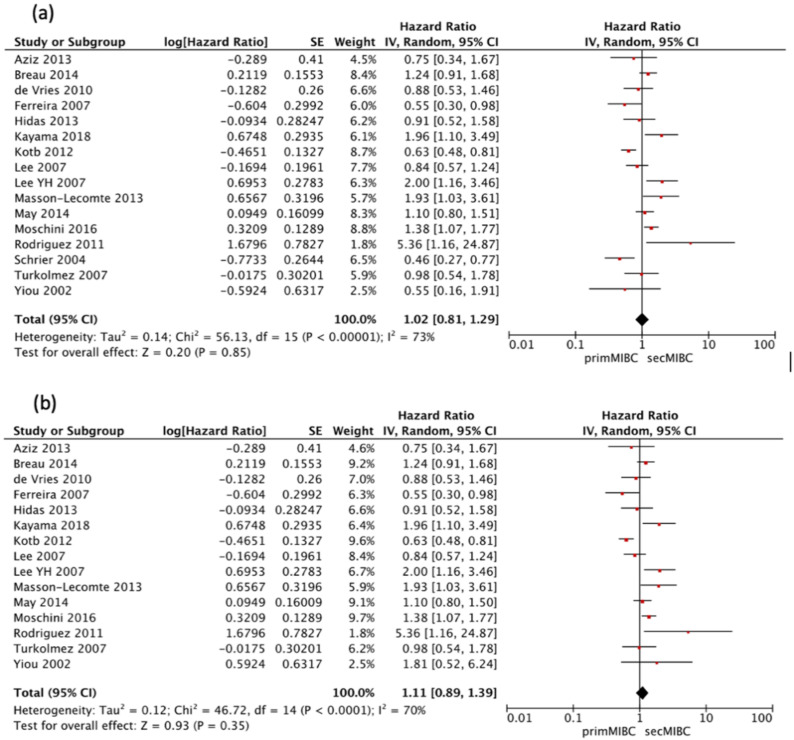
(**a**) Forest Plot for 5-year CSS according to starting point of follow-up, (**b**) Forest Plot for 5-year CSS after RC.

Sixteen studies [4,6,7,8,9,10,11,12,13,14,15,16,17,18,23,24] were available for HR calculation of 5-year CSS between primMIBC and secMIBC. Pooled analysis showed no significant difference in 5-year CSS (pooled HR 1.02, 95%CI 0.81–1.29, *p* = 0.85). However, there was significant heterogeneity between studies (Chi^2^ = 56.13, *I*^2^ = 73%, *p* < 0.001) (Figure 3a).

All of the included studies started follow-up at the time of RC except the one by Schrier et al. [6], which defined the starting point of follow-up from the time of muscle-invasiveness. Hence a separate analysis excluding this study was performed. Pooled analysis showed no significant differences in 5-year CSS (pooled HR 1.11, 95% CI 0.89–1.39, *p* = 0.35), with significant heterogeneity between studies (Chi^2^ = 46.72, *I*^2^ = 70%, *p* < 0.001) (Figure 3b).

**Figure 4:**
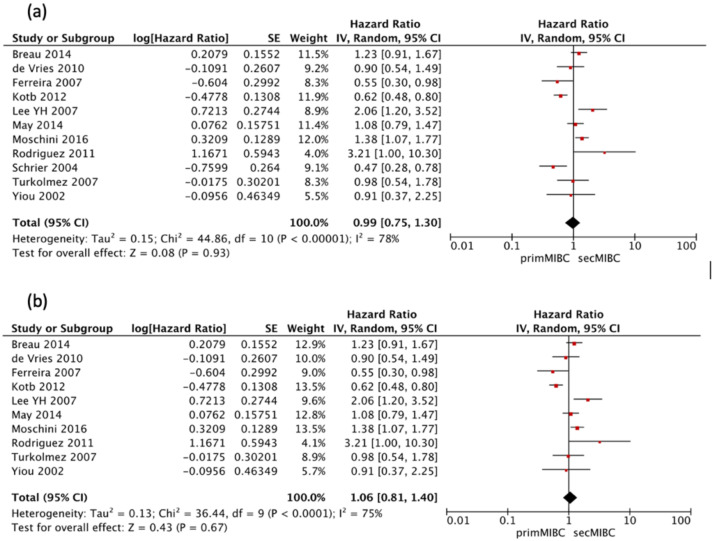
(**a**) Forest Plot for 10-year CSS according to starting point of follow-up, (**b**) Forest Plot for 10-year CSS after RC.

Eleven studies [4,6,7,8,9,12,13,15,16,17,24] were available for HR calculation of 10-year CSS between primMIBC and secMIBC. Pooled analysis showed no significant differences in 10-year CSS (pooled HR 0.99, 95%CI 0.75–1.3, *p* = 0.93), with significant heterogeneity between studies (Chi^2^ = 44.86, *I*^2^ = 78%, *p* < 0.001) (Figure 4a).

Once more, all included studies defined follow-up at the time of RC except the one by Schrier et al. [6]. Hence a separate analysis excluding this study was performed. Pooled analysis showed no significant differences in 10-year CSS (pooled HR 1.06, 95%CI 0.81–1.4, *p* = 0.67), with significant heterogeneity between studies (Chi^2^ = 36.44, *I*^2^ = 75%, *p* < 0.001) (Figure 4b).

**Figure 5:**
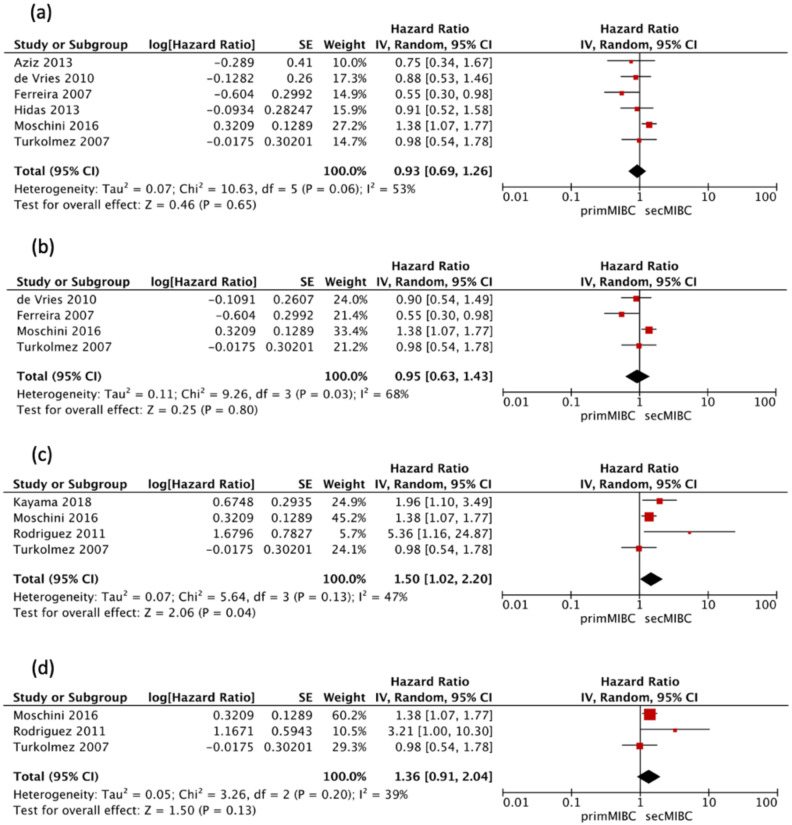
(**a**) Forest plot 5-year CSS including only studies utilizing second-look TURBT, (**b**) Forest plot 10-year CSS including only studies utilizing second-look TURBT, (**c**) Forest 5-year CSS including only studies utilizing NAC, (**d**) Forest plot 10-year CSS including only studies utilizing NAC.

Six studies [7,10,12,13,14,15] reported a second look TURBT and/or presence of detrusor-muscle in the initial TURBT specimen. Pooled analysis of this studies showed no significant differences in the 5-year CSS (pooled HR 0.93, 95% CI 0.69–1.26, *p* = 0.65). There was no significant heterogeneity between studies (Chi^2^ = 10.63, *I^2^* = 53%, *p* = 0.06) (Figure 5a).

Four of these studies [7,12,13,15] were available for HR calculation of 10-year CSS between primMIBC and secMIBC. Pooled analysis showed no significant differences in 10-year CSS (pooled HR 0.95, 95% CI 0.63–1.43, *p* = 0.8), with significant heterogeneity between studies (Chi^2^ = 9.26, *I*^2^ = 68%, *p* = 0.03) (Figure 5b).

Four studies [7,11,12,24] that utilized neoadjuvant chemotherapy (NAC) were available for HR calculation of 5-year CSS (model VI) between primMIBC and secMIBC. Pooled analysis showed significant differences in 5-year CSS between studies (pooled HR 1.5, 95% CI 1.02–2.2, *p* = 0.04), without significant heterogeneity between studies (Chi^2^ = 5.64, *I*^2^ = 47%, *p* = 0.13) (Figure 5c).

Three of these studies [7,12,24] were available for HR calculation of 10-year CSS between primMIBC and secMIBC. Pooled analysis showed no significant differences in 10-year CSS (pooled HR 1.36, 95% CI 0.91–2.04, *p* = 0.13), without significant heterogeneity between studies (Chi^2^ = 3.26, *I*^2^ = 39%, *p* = 0.2) (Figure 5d).

### 3.5. Progression Free Survival

**Figure 6:**
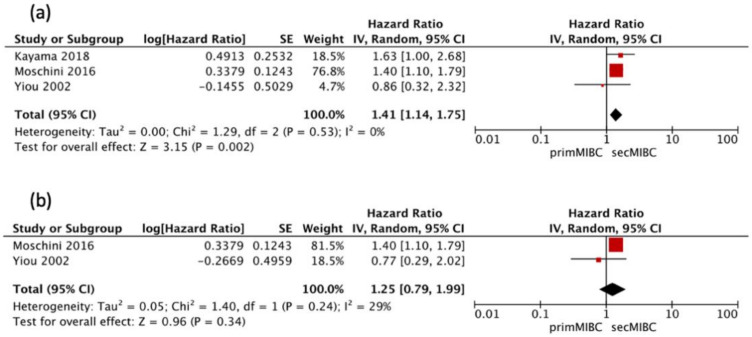
(**a**) Forest Plot PFS 5-year after RC, (**b**) Forest Plot PFS 10-year after RC.

Three studies [7,11,16] were available for HR calculation of 5-year PFS between primMIBC and secMIBC. Pooled analysis showed significant differences in 5-year PFS (pooled HR 1.41, 95% CI 1.14–1.75, *p* = 0.002) without significant heterogeneity between studies (Chi^2^ = 1.29, *I^2^* = 0%, *p* = 0.53) (Figure 6a).

Two of these studies [7,16] were available for HR calculation of 10-year PFS between primMIBC and secMIBC. Pooled analysis showed no significant differences in 10-year PFS (pooled HR 1.25, 95% CI 0.79–1.99, *p* = 0.34), without significant heterogeneity between studies (Chi^2^ = 1.40, *I^2^* = 29%, *p* = 0.24) (Figure 6b).

### 3.6. Quality Assessment

Quality assessment and scores according to the Newcastle-Ottawa Scale are shown in Table 3. All studies had a score of ≥7 and were, therefore, considered to be of high quality.

### 3.7. Publication Bias

**Figure 7:**
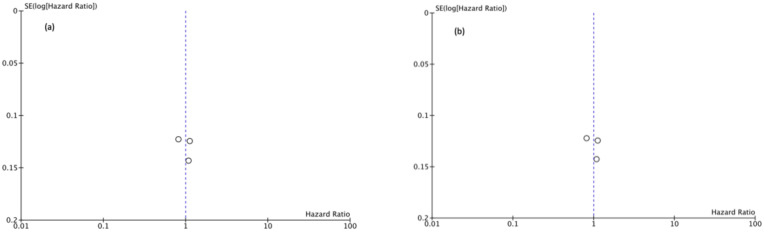
(**a**) Funnel Plot OS 5y, (**b**) Funnel Plot OS 10y.

**Figure 8:**
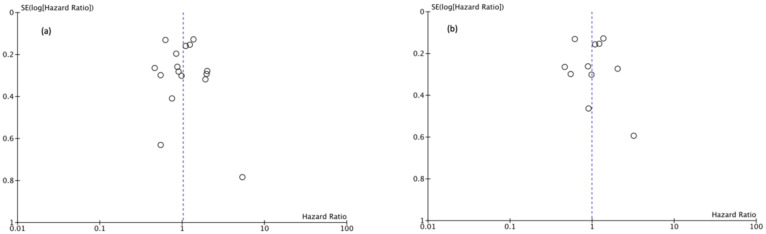
(**a**) Funnel Plot CSS 5y, (**b**) Funnel Plot CSS 10y.

**Figure 9:**
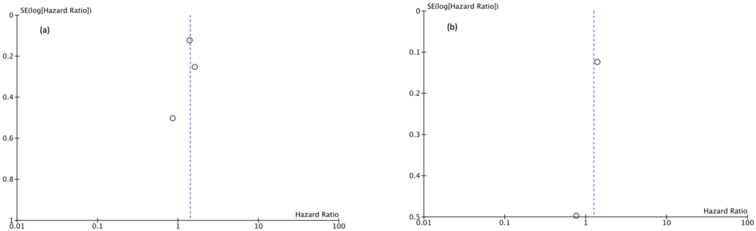
(**a**) Funnel Plot PFS 5y, (**b**) Funnel Plot PFS 10y.

Funnel plots showed asymmetry indicating the presence of a publication bias (Figure 7, Figure 8 and Figure 9).

## 4. Discussion

This systematic review and meta-analysis assessed the differences in oncologic outcomes between primMIBC and secMIBC. 

Two systematic reviews and meta-analyses on this topic have been previously conducted [25,26]. Chen et al. reported similar results to ours, while Ge et al. found a statistically significant worse survival in patients with secMIBC in comparison to those with primMIBC. Both studies suffered from limitations such as incompleteness in studies retrieved. Chen et al. failed to identify three studies in their analyses [10,11,23], and Ge et al. also overlooked three studies [9,11,13]. Chen et al. only provided the extracted data to calculate the HR for CSS from six studies [6,7,8,9,15]. They reported 5-year CSS follow-up data of eight studies [4,6,7,8,9,12,15] and the 10-year follow-up of three studies [7,9,16] accounting for different follow-up times by calculating the OR but not the HR. Ge et al. provided the HR for CSS of thirteen studies but did not account for the different follow-up times by comparing the 5-year HR for CSS [10,14,18] with the 10-year HR [4,6,7,8,12,15,16,17]. Both systematic reviews did not provide data on PFS and response to neoadjuvant chemotherapy. 

We expanded upon these studies and accounted for the different follow-up times by extracting the 5-year and 10-year follow-up data for OS, CSS and PFS. We found similar outcomes regarding OS and CSS between patients with primMIBC compared to those with secMIBC. One possible explanation for this is the vigorous surveillance regimen patients with secMIBC receive before developing muscle-invasive cancer compared to patients with primMIBC, which allows early detection of muscle-invasive status and timely provision of appropriate treatments such as RC. In theory this would offer patients the benefit of keeping a functional bladder while providing a potential survival benefit because of early tumor detection.

Non-muscle-invasive bladder cancer constitutes a heterogenous population regarding biological characteristics, clinical behavior and outcomes. A major part of NMIBC cases can be treated by TURBT and intravesical instillations as the mainstay of treatment in a curable setting. Despite these treatments a proportion of patients progresses to muscle-invasive status. Currently RC, with NAC in eligible patients, is considered the standard of care in the management of MIBC. Multiple studies demonstrated that a delay in RC in MIBC patients worsens prognosis [27,28,29,30]. So naturally the question arises if patients with NMIBC have the same prognosis than those with MIBC because of delayed RC [14,31]. The reported incidence of delayed RC in the literature ranges from 12 to 29% for secMIBC but only from 6 to 13% for primMIBC [14,31], offering one possible explanation for equal prognosis between the two groups. These data is supported by Moschini et al. [7], who concluded that when bladder cancer is still at the NMIBC stage a risk sub-stratification is needed because a subgroup of patients with NMIBC will possible gain a prognostic advantage from RC. Nevertheless, some urologists still oppose to upfront RC in the case of high-risk NMIBC due to the unneglectable morbidity and mortality of associated with RC [32,33] and comparable higher quality of life with bladder sparring treatment strategies [34,35]. 

A subgroup analysis only including those studies utilizing second-look TURBT or reporting the detection of detrusor muscle in TURBT sample showed again no difference in 5-year CSS (HR 0.93, 95% CI 0.69–1.26) and 10-year CSS (HR 0.95, 95% CI 0.63–1.43). Up-staging at second look TURBT can occur in up to 30% of cases, with re-TURBT allowing for more accurate tumor-staging and therefore avoiding inadequate treatment [36,37]. Furthermore, residual tumor is common after TURBT for high-risk NMIBC [37]. Only six [7,10,12,13,14,15] of the sixteen included studies stated utilization of re-TURBT or reported the detection of detrusor-muscle in the initial TURBT sample as quality indicators in patients initially diagnosed with T1 disease, which suggests the potential understaging and inaccurate initial diagnosis. This probably diminished the prognosis of secMIBC to that of primMIBC. 

In addition to primary and secondary patterns of muscle-invasiveness fundamental genetic and molecular mechanisms of tumor induction, promotion and progression necessitate consideration. Intravesical immunotherapy with Bacille Calmette-Guérin (BCG) as well as intravesical chemotherapy and systemic cytotoxic therapy may lead to selection and proliferation of cancer stem cells and a copiousness of these clones. This hypothesis that cancer stem cells play a role in urothelial bladder cancer progression is supported by basic research studies [38,39]. Possible explanations for this include a reduced growth rate, an increase in DNA repair mechanisms [40], creation of a tumor-micro-environment that restricts drug penetration [41], as well as an increase in the ability to efflux drugs from the cell [42]. 

This is backed up by a recent study from Pietzak et al. [20], who showed that patients with secMIBC treated with NAC had worse oncologic outcomes compared to patients with primMIBC. Moreover, they found more deleterious somatic *ERCC2* missense mutations, resulting in an increase in cisplatin sensitivity, in chemotherapy-naïve primMIBC compared to secMIBC. Due to the lack of cisplatin-based NAC they concluded that patients with secMIBC should undergo upfront RC or enrollment in clinical trials. This generates the hypothesis, of a genomic based differential response to neoadjuvant chemotherapy in primMIBC compared to secMIBC. Apart from this study there is an astonishing lack of publications investigating the response for NAC via biomarkers between primMIBC and secMIBC. In our meta-analysis, the use of neoadjuvant chemotherapy was only reported in six [7,9,11,12,24] of the sixteen included studies with a low proportion of patients receiving this treatment. A subgroup analysis of these studies showed a statistically significant better 5-year CSS in primMIBC and trend favoring primMIBC for 10-year CSS (HR 1.36, 95% CI 0.91–2.04). 

We found that secMIBC is associated with a significantly worse 5-year, but not 10-year PFS; however only three studies were available for analysis.

The heterogeneity in the secMIBC awakes the need for tailored treatment approaches. Despite similar differential oncologic outcomes between primMIBC and secMIBC after performance of RC in our recent review, it does not imperatively imply that RC should be postponed until muscle-invasiveness developed in the whole cohort of NMIBC patients, particulary in those showing high-risk features. This is emphasized by de Vries et al. [15]., who stratified secMIBC patients according to the EAU risk categories low/intermediate-risk and high-risk groups. They reported that MIBC resulting from high-risk NMIBC had a worse prognosis than that resulting from low/intermediate risk tumors. May et al. [17] and Aziz et al. [14] demonstrated worse CSS for secMIBC patients with higher EORTC scores further affirming that the performance of RC should not be delayed in secMIBC who developed muscle-invasive cancer in the subpopulation of high-risk NMIBC. All these data suggest significant variablitiy in oncological outcomes in patients with secMIBC. Breau et al. [4] and May et al. [17] compared the prognosis of primMIBC, secMIBC and high-risk NMIBC and showed better survival outcomes after RC for the latter.

The contemporaneous risk classification for bladder cancer includes tumor stage, tumor size, tumor grade, multifocality, presence of lympho-vascular invasion (LVI) and presence of carcinoma in situ (CIS) [3]. Prospective clinical trials incorporating genetic and molecular drivers for progression to muscle-invasiveness are needed to develop risk stratification tools that can sufficiently differentiate between NMIBC patients benefiting from up-front RC and those benefiting from bladder preserving strategies.

We believe our systematic review and meta-analysis offers new insights into the question whether oncological outcome between primMIBC and secMIBC differs. However, caution should be exercised in interpreting the conclusion drawn from this study given the limitations, which include the retrospective nature of the primary data included and the potential selection bias. The publishing date of individual studies ranged from 2002 to 2018 and the resulting changes in treatment approaches over time may have influenced the results (e.g., implementation of re-TURBT). Another weakness of our study was heterogeneity of the included studies. Even though heterogeneity was accounted for by applying a random effect model, individual publications have differed with regarding to baseline data of included patients, surgical techniques, follow-up schemes and implementation of bladder preserving strategies. For quality assessment of the included publications the NOS was used but has to be interpreted with caution because no standard validated end point criteria have been defined for its usage. Therefore, we considered studies scoring seven or more stars as high quality. Even though the NOS is commonly applied in evidence-based systematic reviews and meta-analysis its use remains controversial and it was shown to produce highly inconsistent results [43,44]. To decrease a possible bias by including publications of low quality we applied rigorous criteria for inclusion and exclusion of a study into our review and data extraction.

## 5. Conclusions

This systematic review and meta-analysis showed similar OS and CSS between patients with primMIBC and secMIBC treated with RC. The worse outcomes for patients with secMIBC treated with neoadjuvant chemotherapy, generate the hypothesis that there is a potential need for RC first in secMIBC versus the present standard of care that is NAC with subsequent RC if possible. Prospective trials incorporating genetic and molecular drivers for progression to muscle-invasiveness are necessary to develop novel risk stratification tools that can be used to differentiate between patients with NMIBC requiring upfront RC and those who can be managed with bladder preserving strategies. 

## Figures and Tables

**Table 1:** Characteristics of included studies.

Study	Country	Study Design	No. of Patients	Prim	Sec	Duration	Mean FU (Months)	Start of Follow-Up	Surveillance Time Prim (Months)	Treatment of Sec
Yiou [16]	France	Retrospective	55	43	12	1987–1997	prim: 49, sec: 55.3	RC	57	TURBT BCG
Schrier [6]	Netherlands	Retrospective	163	89	74	1986–2000	NA	MIBC	NA	TURBT BCG
Ferreira [13]	Brazil	Retrospective	242	185	57	1993-2005	prim: 98, sec: 96	RC	37.4	TURBT BCG
Lee YH [8]	Korea	Retrospective	223	173	50	1986–2004	45	RC	15	TURBT BCG
Turkolmez [12]	Turkey	Retrospective	154	109	45	1990–2005	prim: 77.8, sec: 90.3	RC	41.7	TURBT BCG
Lee [18]	USA	Retrospective	239	169	70	1990–2003	prim: 40, sec: 33 (median)	RC	48	TURBT BCG
de Vries [15]	Netherlands	Retrospective	188	134	54	1987–2005	40.8	RC	NA	TURBT
Rodriguez [24]	Spain	Retrospective	141	72	69	1978–2002	42.5	RC	NA	TURBT
Kotb [9]	Canada	Retrospective	1150	785	365	NA	NA	RC	NA	NA
Masson-Lecomte [23]	France	Retrospective	179	155	24	2001–2011	NA	RC	36	TURBT BCG
Hidas [10]	Israel	Retrospective	144	104	40	1998–2008	prim: 40.1, sec: 52.6	Initial TURB+RC	44	TURBT BCG
Aziz [14]	Germany	Retrospective	150	125	25	2004–2010	46 (median)	RC	17.71	TURBT BCG
May [17]	Germany	Retrospective	521	399	122	1992–2007	65	RC	21.72	TURBT BCG
Breau [4]	Canada	Retrospective	671	481	190	1980–1998	NA	RC	21.6	TURBT BCG
Moschini [7]	Italy	Retrospective	768	475	293	2000–2012	109	RC	NA	TURBT BCG
Kayama [11]	Japan	Retrospective	282	231	51	2004-2015	25–161	RC	NA	NA

MIBC: Muscle invasive bladder cancer, Prim: primary MIBC, sec: secondary MIBC, RC: radical cystectomy, TURBT: transurethral resection of the bladder tumor, BCG: Bacille Calmette-Guérin, NA: not available.

**Table 2:** Characteristics of included patients.

Study	Mean Age (Years)		Gender (Male) n (%)		Tumor Stage at RC n (%)				HG n (%)		CIS n (%)		LVI n (%)	
	Prim	Sec	Prim	Sec	T3/4		N+		Prim	Sec	Prim	Sec	Prim	Sec
					Prim	Sec	Prim	Sec						
Breau [4]	67.9	67.6	366 (76)	146 (77)	194 (40)	96 (51)	100 (21)	38 (20)	NA	NA	NA	85 (45)	NA	NA
Schrier [6]	63.3	68.5	65 (73)	60 (81)	NA	NA	27 (30)	21 (28)	NA	NA	NA	NA	NA	NA
Moschini [7]	68	67	319 (82)	250 (85)	292 (61)	195 (67)	NA	NA	400 (84)	263 (90)	96 (20)	53 (18)	112 (24)	91 (31)
Lee YH [8]	62		154 (89)	46 (92)	76 (44)	26 (52)	26 (15)	14 (28)	155 (90)	40 (80)	35 (20)	10 (20)	38 (22)	12 (24)
Kotb [9]	NA		623 (80)	291 (80)	451 (58)	131 (36)	NA	NA	697 (91)	338 (97)	NA	NA	254 (46)	78 (32)
Hidas [10]	72.7	69.3	79 (76)	33 (83)	30 (47)	14 (62)	13 (20)	2 (9)						
Kayama [11]	71 (31–91) (median)		188 (81)	40 (78)	82 (36)	22 (43)	0 (0)	0 (0)	117 (77)	36 (71)	20 (9)	8 (16)	83 (36)	18 (35)
Turkolmez [12]	59.8	60.3	94 (86)	40 (89)	48 (44)	20 (44)	NA	NA	NA	NA	NA	NA	NA	NA
Ferreira [13]	65.3	63.7	145 (78)	47 (83)	80 (43)	28 (49)	57 (21)	16 (28)	NA	NA	NA	NA	NA	NA
Aziz [14]	69	71	97 (78)	24(96)	76 (61)	17 (68)	50 (40)	9 (36)	114 (91)	25 (100)	61 (49)	11 (44)	72 (58)	13 (52)
de Vries [15]	61		103 (77)	41 (76)	42 (31)	13 (24)	60 (45)	25 (46)	NA	NA	NA	NA	NA	NA
Yiou [16]	62	66	NA	NA	25 (58)	3 (25)	13 (30)	21 (6)	29 (67)	6 (50)	NA	NA	NA	NA
May [17]	64.1	68.7	388/133		138 (56)	52 (57)	88 (36)	28 (30)	178 (72)	62 (68)	NA	NA	NA	NA
Lee [18]	65	69	127 (75)	55 (79)	93 (55)	41 (61)	46 (28)	15 (22)	161 (96)	65 (93)	NA	NA	NA	NA
Masson-Lecomte [23]	66.8	68	166/25		NA	NA	46 (30)	11 (42)	NA	NA	NA	NA	NA	NA
Rodriguez [24]	63 (median)		116/25		NA	NA	NA	NA	NA	NA	NA	NA	NA	NA

N+: metastasis in a single or multiple lymph nodes in the true pelis or in common iliac lymph nodes, HG: high grade, CIS: carcinoma in situ, LVI: lymphovascular invasion.

**Table 3:** Newcastle-Ottowa Scale quality assessment.

First Author	Year	Selection	Comparability	Outcome	Total Score
Breau [4]	2014	4	2	3	9
Schrier [6]	2004	4	1	3	8
Moschini [7]	2016	4	2	3	9
Lee YH [8]	2007	4	1	3	8
Kotb [9]	2012	4	0	3	7
Hidas [10]	2013	4	2	3	8
Kayama [11]	2018	3	1	3	7
Turkolmez [12]	2007	3	1	3	7
Ferreira [13]	2007	4	1	3	8
Aziz [14]	2013	3	1	3	7
de Vries [15]	2010	4	1	3	8
Yiou [16]	2002	2	2	3	7
May [17]	2014	4	2	3	9
Lee [18]	2007	4	2	3	9
Masson-Lecomte [23]	2013	3	1	3	7
Rodriguez [24]	2011	3	1	3	7

## Data Availability

The data presented in this article are available in [4,6,7,8,9,10,11,12,13,14,15,16,17,18,23,24].
